# Prevalence of Echocardiography Use in Patients Hospitalized with Confirmed Acute Pulmonary Embolism: A Real-World Observational Multicenter Study

**DOI:** 10.1371/journal.pone.0168554

**Published:** 2016-12-15

**Authors:** Rong Bing, Vincent Chow, Jerrett K. Lau, Liza Thomas, Leonard Kritharides, Austin Chin Chwan Ng

**Affiliations:** 1 Cardiology Department, Concord Hospital, The University of Sydney, Concord, New South Wales, Australia; 2 Cardiology Department, Liverpool Hospital, Sydney South West Clinical School, University of New South Wales, Liverpool, New South Wales, Australia; Azienda Ospedaliero Universitaria Careggi, ITALY

## Abstract

**Background:**

Acute pulmonary embolism (PE) carries an increased risk of death. Using transthoracic echocardiography (TTE) to assist diagnosis and risk stratification is recommended in current guidelines. However, its utilization in real-world clinical practice is unknown. We conducted a retrospective observational study to delineate the prevalence of inpatient TTE use following confirmed acute PE, identify predictors for its use and its impact on patient’s outcome.

**Methods:**

Clinical details of consecutive patients (2000 to 2012) from two tertiary-referral hospitals were retrieved from dedicated PE databases. All-cause and cause-specific mortality was tracked from a state-wide death registry.

**Results:**

In total, 2306 patients were admitted with confirmed PE, of whom 687 (29.8%) had inpatient TTE (39.3% vs 14.4% between sites, P<0.001). Site to which patient presented, older age, cardiac failure, atrial fibrillation and diabetes were independent predictors for inpatient TTE use, while malignancy was a negative predictor. Overall mortality was 41.4% (mean follow-up 66.5±49.5months). Though inpatient TTE use was not an independent predictor for all-cause or cardiovascular mortality in multivariable analysis, in the inpatient TTE subgroup, right ventricle-right atrial pressure gradient (hazard ratio [HR] 1.02 per-1mmHg increase, 95% confidence interval [CI] 1.01–1.03) and moderate/severe aortic stenosis (HR 2.26, 95% CI 1.20–4.27) independently predicted all-cause mortality.

**Conclusions:**

Inpatient TTE is used infrequently in real-world clinical settings following acute PE despite its usefulness in risk stratification, prognostication and assessing comorbid cardiac pathologies. Identifying patients that will benefit most from a TTE assessment following an acute PE episode and reducing barriers in accessing TTE should be explored.

## Introduction

Acute pulmonary embolism (PE) is the most serious complication of venous thromboembolism (VTE) with a reported annual incidence of 100–200 per 100,000 persons [1]. In-hospital mortality varies widely depending on thrombotic burden but can be as high as 59%.[2] Long-term morbidity and mortality remains high [3], with a reported 4-year adverse event rate of 50% [4] and a 5-year mortality rate of up to 32% [5].

Although transthoracic echocardiography (TTE) can detect features of right ventricular (RV) strain from massive and submassive PE, it cannot be used to reliably diagnose PE [6, 7]. The detection of RV dysfunction using TTE in patients with known PE, however, has been linked to outcomes, even in the absence of hemodynamic instability [8–11]. The American Heart Association guideline recognizes the role of TTE in identifying patients at increased risk of adverse outcomes, although no specific level of recommendation is provided [12]. Assessment of the RV with TTE receives a class-IIa recommendation in the European Society of Cardiology guideline for risk stratification of intermediate risk patients with acute PE [13]. There is no dedicated Australian guideline on the use of TTE for acute PE. Contemporary “real-world” prevalence of TTE use following hospital admission for confirmed acute PE has not been studied to date. Moreover, the impact on outcomes arising from whether a patient received a TTE or not during the index PE admission is not known.

We conducted a retrospective observational study with the primary aim of determining the prevalence of inpatient TTE use in a large multicenter cohort of patients with radiologically confirmed PE managed in a real-world clinical setting. Secondary aims of our study include identifying predictors for inpatient TTE use and determining if the use of inpatient TTE, its timing, or any reported echocardiographic parameters had an impact on all-cause and cardiovascular mortality.

## Materials and Methods

### Study cohorts

The primary PE cohort was derived from a university-affiliated tertiary institution’s PE database (Concord Repatriation General Hospital [CRGH], New South Wales [NSW], Australia) maintained by its Cardiology Department. Outcomes of patients with confirmed PE from this database have been reported [5, 14, 15]. For the purpose of this study, consecutive patients admitted with a primary diagnosis of acute PE between January 2000 and December 2012 were identified. All patients’ medical records were reviewed to confirm the diagnosis of PE as per guideline [13], requiring both a documented clinical diagnosis and/or treatment of acute PE, together with either an intermediate-high probability nuclear pulmonary ventilation-perfusion scintigraphy [V/Q scan] for PE or computed-tomography pulmonary angiogram [CTPA] showing thrombus within the pulmonary arterial circulation.

A second cohort was identified from a separate university-affiliated tertiary institution’s PE database (Liverpool Hospital [LH], NSW, Australia). This institution is located in a different demographic metropolitan area. The outcomes of these patients have also been reported [16]. For the current study, consecutive patients admitted with a primary diagnosis of acute PE between January 2000 and December 2010 were identified. The same criteria as described above were used to confirm the diagnosis of PE.

Patients from either cohort that presented on more than one occasion with acute PE during the study period only had their index admission included. Patients from other states or countries were excluded to optimize the completeness of follow-up.

The study was conducted according to the principles expressed in the Declaration of Helsinki, and was approved by both institutions’ Human Research Ethics Committees (CRGH Research Ethics Committee: CH62/6/2015-062; LH Research Ethics Committee: SSA/11/LPOOL/112). Both Committees also granted a waiver for consent from the individual to the use of their health information. The study had a retrospective design and patients’ data were de-identified and analyzed anonymously.

### Data collection

Patients’ demographics, comorbidities and in-hospital outcomes were retrieved from the PE databases, in addition to the use and timing of inpatient TTE during the index PE admission. Early inpatient TTE was defined as occurring on day-0 or day-1 of admission. The echocardiography service at both institutions has been established for many years, with an average of 4000–5000 TTEs performed per annum at each site. In both institutions, whether a patient receives an inpatient TTE assessment following admission for an acute PE is at the discretion of the treating physician. Comorbidities were grouped based on the International Classification of Diseases (ICD-10). Cardiovascular disease (CVD) was defined as a composite of ischemic heart disease, congestive cardiac failure, atrial fibrillation/flutter, valvular heart disease, stroke and peripheral vascular disease. Cardiac risk factors of hypertension, dyslipidemia, diabetes and current or previous smoking history were recorded separately and as a combined variable. The Charlson Comorbidity Index (CCI) was used to assess the overall comorbidity burden of each patient [17]. The index was recorded as a continuous variable and separately stratified into three tiers (0, 1–2 and ≥3).

All TTEs were performed by qualified sonographers and reported by experienced cardiologists in both institutions. Information such as left ventricular (LV) function, dilated RV and/or RV function impairment, right ventricle-right atrial (RV-RA) pressure gradient and major valvular disease were extracted from finalized reports and stratified in a standardized fashion. There was no attempt to verify the reports. For the purpose of this study, the RV-RA pressure gradient was chosen (derived from the modified Bernoulli equation 4(V)^2^, where V is the peak velocity [in meters per second] of the tricuspid valve regurgitant jet)[18] and not pulmonary artery systolic pressure as inferior vena cava collapsibility was not consistently reported in a large number of patients who had TTE.

The CRGH database contains additional details of patients’ hemodynamic and blood profiles (not recorded in the LH cohort). This allowed the calculation of the simplified Pulmonary Embolism Severity Index (sPESI) score for the CRGH cohort [19, 20]. The index is derived from a patient’s age, sex, history of malignancy, heart failure or chronic lung disease, heart rate (≥110beats/minute), systolic blood pressure (<100mmHg) and arterial oxyhemoglobin saturation (<90%). In addition, we calculated each patient’s shock index, derived from heart rate divided by systolic blood pressure, with a score >0.7 signifying hemodynamic compromise [21].

### Study outcomes

The primary outcome was the prevalence of inpatient TTE use following confirmed acute PE. Secondary outcomes included all-cause and cardiovascular mortality following the PE, and whether having an inpatient TTE influenced these outcomes. Census dates for the PE databases were October 2013 and September 2012 for the CRGH and LH cohorts respectively. A state-wide death registry database was used to track all-cause mortality. Using the death registry is advantageous as non-captured deaths during study period were estimated to be only 0.6% based on known migration rates [22]. All death certificates were reviewed to ascertain cause-specific mortality outcomes. Cardiovascular death was defined as death due to PE, acute myocardial infarction, heart failure, stroke, cardiac arrest and cardiac-related causes (when more than one cardiac cause of death was recorded). Each death was coded independently by two authors (A.C.C.N., L.K. or J.K.L) according to the general principles set by the World Health Organization [23]. During the death coding process, patients were de-identified so that researchers were blinded to patients’ background history. Coding disparities were resolved by consensus.

### Statistical analysis

All continuous variables are expressed as mean ± standard deviation; categorical data are given in frequency and percentages. Patients were stratified into those who underwent a TTE during the PE admission and those who did not; and in the subgroup of patients who had TTE, patients were stratified by their study sites (i.e. CRGH versus LH site). Comparisons between two groups were performed using unpaired t test for continuous variables, and χ2 test or Fisher’s exact test for dichotomous variables. Predictors for inpatient TTE use were determined using binary logistic regression analysis. Univariables were based on clinically relevant parameters listed in Table 1. Those with *P*<0.10 were included in the multivariable analysis; age and sex were entered into the modelling regardless of *P* value. Kaplan-Meier survival methods were used to compare unadjusted survival rates. Cox proportional hazards regression analysis was used to assess adjusted survival outcome.

**Table 1:** Patient characteristics during index PE admission.

Admission parameters	Inpatient	No inpatient	Total cohort
	TTE n = 687	TTE n = 1619	n = 2306
Age—years	68.3±15.9 *	63.1±17.5	64.6±17.2
Males—no. (%)	306 (44.5)	713 (44.0)	1019 (44.2)
Length of admission—days	10.1±7.5 *	7.1±6.5	8.0±6.9
Days to inpatient TTE	3.4±6.4	-	-
Early TTE ^†^ –no. (%)	242 (35.2)	-	-
**Imaging—no. (%)**			
V/Q scintigraphy	471 (69.4) *	967 (63.7)	1438 (65.5)
CTPA	270 (40.0)	628 (42.0)	898 (41.4)
Both modalities ^‡^	69 (10.0)	126 (7.8)	195 (8.5)
**Comorbidities—no. (%)**			
Cardiovascular disease	328 (47.8) *	420 (26.0)	748 (32.5)
IHD	138 (20.1) *	197 (12.2)	335 (14.5)
CCF	110 (16.0) *	105 (6.5)	215 (9.3)
Atrial fibrillation/flutter	121 (17.6) *	137 (8.5)	258 (11.2)
Valvular heart disease	20 (2.9)	35 (2.2)	55 (2.4)
Stroke	17 (2.5)	35 (2.2)	52 (2.3)
PVD	72 (10.5) *	107 (6.6)	179 (7.8)
Cardiac risk factors	397 (57.8)	870 (53.7)	1267 (54.9)
Hypertension	202 (29.4) *	387 (23.9)	589 (25.5)
Dyslipidemia	91 (13.2) *	168 (10.4)	259 (11.2)
Diabetes	123 (17.9) *	199 (12.3)	322 (14.0)
Current smoker	64 (9.3) *	232 (14.3)	296 (12.8)
Ex-smoker	120 (17.5)	273 (16.9)	393 (17.0)
Chronic pulmonary disease	76 (11.1)	181 (11.2)	257 (11.1)
Chronic kidney disease	56 (8.2) *	93 (5.7)	149 (6.5)
Malignancy	108 (43.0) *	206 (53.4)	314 (49.3)
**Charlson Comorbidity Index**			
Mean score	1.50±1.79	1.37±1.80	1.41±1.80
≥ 3	151 (22.0)	300 (18.5)	451 (19.6)
1–2	278 (40.5)	592 (36.6)	870 (37.7)
0	258 (37.6) *	727 (44.9)	985 (42.7)

* P<0.05 between inpatient and no inpatient TTE

^†^ Early inpatient TTE was defined as occurring on day-0 or day-1 of the index PE admission.

^‡^ Both modality indicates patient had both V/Q and CTPA performed during index PE admission.

Plus-minus values represent mean ± standard deviation (all others represent numbers of patients with values in brackets representing percentages). IHD, ischemic heart disease; CCF, congestive cardiac failure; PVD, peripheral vascular disease; PE, pulmonary embolism; V/Q, ventilation/perfusion; CTPA, computed tomography pulmonary angiography; TTE, transthoracic echocardiogram.

To determine the impact of inpatient TTE use on mortality, a multivariable model was created (Model 1), adjusted for inpatient TTE use, site (CRGH versus LH), age, sex, and comorbidities including ischemic heart disease, congestive cardiac failure, atrial fibrillation/flutter, stroke, peripheral vascular disease, diabetes, current smoker, chronic pulmonary disease, chronic kidney disease and malignancy. A second multivariable model (Model 2) assessed whether early vs late TTE (day-0 or day-1 vs >day-1), adjusted for site, age, sex and CCI, affected mortality, as previous study suggested performing TTE at day-1 following an acute PE could be prognostically important [24]. To assess the prognostic value of individual echocardiographic parameters, a separate multivariable analysis was performed, adjusted for relevant TTE parameters, site, age, sex and CCI. Finally, supplementary subgroup analyses were performed on the CRGH cohort using the additional clinical information available. The statistical method of case-wise deletion was used to deal with missing data [25]. *P* values <0.05 were considered statistically significant. All analyses were performed using SPSS v22 (IBM Corp, Armonk, NY).

## Results

In total, 2306 consecutive patients with confirmed PE were included in this study: 1426 patients from CRGH, and 880 patients from LH. Data on TTE use was available for all patients: 687 (29.8%) patients underwent inpatient TTE, with a significant difference between CRGH and LH (39.3% vs 14.4% respectively, *P*<0.001). Of those who received an inpatient TTE, the study was performed early, i.e. on day-0 or day-1 of admission, in 242 (35.2%) patients.

### Admission characteristics

Table 1 outlines patient characteristics for the combined cohort. The inpatient TTE cohort were older with significantly higher prevalence of cardiovascular disease and risk factors, but fewer known malignancy. Although mean CCI score was not significantly different, there were fewer patients with a score of 0 in the inpatient TTE group. Length of hospital admission was significantly longer in the inpatient TTE group (10.1±7.5 vs 7.1±6.5days, *P*<0.001). Compared to CRGH, LH patients were younger (S1 Table); consequently there was less established cardiovascular disease but a higher prevalence of active smoking. The mean CCI at each site did not differ between those who had an inpatient TTE and those who did not.

Of the 1426 CRGH patients, those who had an inpatient TTE had a higher mean sPESI (0.96±0.97 vs 0.83±0.86, *P* = 0.008), were more likely to present with syncope (8.6% vs 3.9%, *P*<0.05) and dyspnea (70.9% vs 62.6%, *P*<0.05) (see S2 Table for additional parameters for CRGH patients). In addition, 44 (3.1%) patients had systolic blood pressure <100mmHg, with no difference between those who had an inpatient TTE versus those who did not (20 [3.6%] vs 24 [2.8%] respectively, *P* = 0.43). There were 172 (12.1%) patients with heart rate >110 bpm, and was proportionally more in those patients who had an inpatient TTE compared to those who did not (81 [14.5%] vs 91 [10.5%] respectively, *P* = 0.03). In total, 421 (29.5%) patients had a shock index >0.7. There was no difference in the proportions of patients with shock between those who had an inpatient TTE versus those who did not (180 [32.1%] vs 241 [27.8%] respectively, *P* = 0.09). Troponin was performed more often (68.2% vs 45.8%, *P*<0.05) and was elevated more often (30.2% vs 14.2%, *P*<0.05) in the inpatient TTE group.

### Inpatient echocardiographic characteristics

TTE findings are shown in Table 2: 36.4% of patients had reported RV dilatation; 32.3% had RV impairment. LH patients were more likely to have these findings than CRGH patients. Additionally, although the mean RV-RA pressure gradient was elevated in both groups, the LH cohort had a significantly higher mean RV-RA pressure gradient (46.1±18.2 vs 36.8±14.3mmHg, *P*<0.001).

**Table 2:** Echocardiography results in patients who received inpatient TTE.

Parameters—no. (%)	Total TTE	CRGH	LH
	n = 687	n = 560	n = 127
Left ventricular ejection fraction ≤50%	204 (29.7)	169 (30.2)	35 (27.6)
Right ventricular dilatation	250 (36.4)	193 (34.5) *	57 (44.9)
Impaired right ventricular contractility	222 (32.3)	172 (30.7)	50 (39.4)
RV-RA pressure gradient (mmHg)	38.6±15.2	36.8±14.3 *	46.1±18.2
Left atrial dilatation	333 (48.5)	283 (50.5) *	50 (39.4)
Right atrial dilatation	217 (31.6)	173 (30.9)	44 (34.6)
Valvular lesions (moderate/severe)			
Aortic stenosis	15 (2.2)	12 (2.1)	3 (2.4)
Aortic regurgitation	11 (1.6)	8 (1.4)	3 (2.4)
Mitral stenosis	4 (0.6)	3 (0.5)	1 (0.8)
Mitral regurgitation	53 (7.7)	41 (7.3)	12 (9.4)
Tricuspid regurgitation	101 (14.7)	81 (14.5)	20 (15.7)

* *P*<0.05 between CRGH and LH patients

Plus-minus values represent mean ± standard deviation (all others represent numbers of patients with values in brackets representing percentages). CRGH, Concord Repatriation General Hospital; LH, Liverpool Hospital; RV-RA pressure gradient, right ventricle-right atrial pressure gradient; TTE, transthoracic echocardiogram.

### Predictors for having an inpatient TTE during admission for acute PE

Multivariable independent predictors for having an inpatient TTE were increasing age, congestive cardiac failure, atrial fibrillation/flutter, diabetes and CRGH as the treating site (see S3 and S4 Tables for univariable and multivariable predictors for having an inpatient TTE respectively). Malignancy was a strong negative predictor.

### Impact of having an inpatient TTE on survival

**Fig 1:**
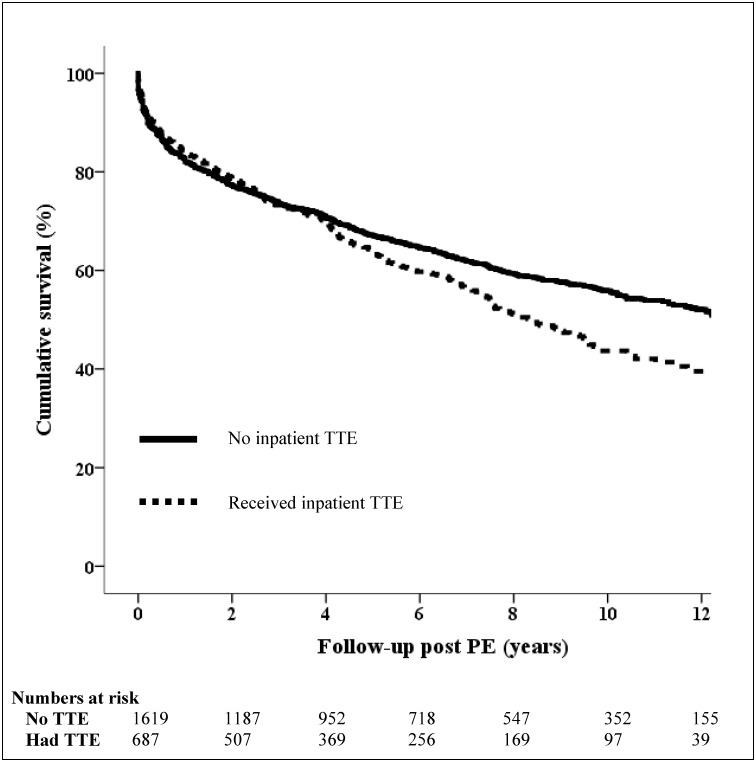
Unadjusted Kaplan-Maier survival curves between patients who received an inpatient TTE versus no TTE during PE admission (combined cohort). The unbroken line represents patients who did not receive an inpatient transthoracic echocardiogram (TTE).The broken line represents patients who had an inpatient TTE during the admission for acute pulmonary embolism (PE). The curves differed significantly for the study period (*P* = 0.005).

Unadjusted Kaplan-Meier survival for the combined cohort comparing inpatient and no inpatient TTE use is presented in Fig 1. Overall mortality was 41.4% at a mean follow-up of 66.5±49.5months. Mean survival time was significantly shorter in patients who had an inpatient TTE (96.0 [95% CI 90.6–101.5] months vs 106.1 [95% CI 102.6–109.6] months, *P* = 0.005) (see S5 Table for mortality rates at various time-points for the two sites). All-cause mortality was numerically greater in the inpatient TTE group, while cardiovascular mortality was significantly higher (18.2% vs 14.3%, *P* = 0.03). This was driven by a higher all-cause mortality rate in the LH inpatient TTE group compared to CRGH (44.9% vs 34.4%, *P* = 0.02) and a non-significant trend towards higher cardiovascular mortality rates at both sites (see Figure in S1 and S2 Figs).

Of the total 2205 patients who survived to discharge in both sites, 853 patients died during the follow-up period (CRGH site, 592 died out of 1381 patients; LH site, 261 died out of 824 patients). A total of 36 deaths were attributed to recurrent PE (CRGH site, 22 [3.7%] out of 592 deaths; LH site, 14 [5.4%] out of 261 deaths), with no significant difference between the two sites (*P* = 0.27). There was also no difference between those patients who received an inpatient TTE versus those who did not received one (inpatient TTE cohort, 16 [5.7%] out of 281 deaths; no inpatient TTE cohort, 20 [3.5%] out of 571 deaths, *P* = 0.15).

After adjusting for site and relevant comorbidities (see S6 Table for univariable analysis), neither the use (Model 1) nor timing (Model 2) of inpatient TTE had an impact on all-cause or cardiovascular mortality (Table 3). As expected, variables such as age, male gender, cardiac failure, chronic kidney disease, malignancy, and CCI retained their prognostic value (see S7 and S8 Tables). In the CRGH cohort where sPESI and troponin levels were known and could be accounted for in multivariable analysis, inpatient TTE was an independent predictor for all-cause mortality (HR 0.84, 95% CI 0.71–0.99, *P* = 0.04), but not for cardiovascular mortality (HR 0.87, 95% CI 0.66–1.14, *P* = 0.31) (see S9 Table).

**Table 3:** Multivariable independent predictors of mortality.

	All-cause mortality		Cardiovascular mortality	
	HR (95% CI)	*P* value	HR (95% CI)	*P* value
**Model 1** (inpatient TTE use) *	1.00 (0.87–1.16)	0.98	0.99 (0.78–1.25)	0.92
**Model 2** (early vs late TTE) ^‡^	1.07 (0.83–1.37)	0.61	1.15 (0.79–1.67)	0.47
**TTE predictors of mortality** ^#^				
LV ejection fraction ≤50%	0.80 (0.59–1.10)	0.17	0.99 (0.62–1.61)	0.98
RV dilatation ^†^	0.88 (0.62–1.25)	0.49	-	-
Impaired RV contractility	1.33 (0.94–1.88)	0.11	1.47 (0.93–2.32)	0.10
RV-RA pressure gradient– 1mmHg increase	1.02 (1.01–1.03)	<0.001	1.01 (1.00–1.03)	0.06
LA dilatation	0.98 (0.72–1.34)	0.91	1.29 (0.78–2.14)	0.31
RA dilatation	1.22 (0.89–1.68)	0.21	1.06 (0.65–1.72)	0.82
Valvular lesions (moderate/severe)				
Aortic stenosis	2.26 (1.20–4.27)	0.01	2.75 (1.10–6.83)	0.03
Mitral regurgitation	0.86 (0.56–1.30)	0.47	1.00 (0.57–1.76)	0.99
Tricuspid regurgitation	1.07 (0.74–1.54)	0.72	0.96 (0.56–1.64)	0.87

* Model 1 analyzed all patients (n = 2306) and adjusted for those variables listed in Methods (see S7 Table for adjusted variables).

^‡^ Model 2 analyzed all patients who underwent inpatient TTE (n = 687) and adjusted for those variables listed in Methods (see S8 Table for adjusted variables).

^#^ This analysis included all patients who underwent inpatient TTE (n = 687), and adjusted for site, age, sex, Charlson Comorbidity Index and echocardiographic univariables with *P*<0.10 (see S10 Table for echocardiographic univariables).

^†^ RV dilatation was not a predictor for cardiovascular mortality in univariable analysis (see S10 Table).

HR, hazard ratio; CI, confidence interval; LV, left ventricle; RV, right ventricle; LA, left atrium; RA, right atrium; TTE, transthoracic echocardiogram.

### Independent predictors of mortality based on echocardiographic parameters

Table 3 shows the independent echocardiographic parameters that predicted mortality for the subgroup who underwent inpatient TTE. RV-RA pressure gradient (HR 1.02 per-1mmHg increase, 95% CI 1.01–1.03) and moderate/severe aortic stenosis (HR 2.26, 95% CI 1.20–4.27) predicted all-cause death. Only moderate/severe aortic stenosis was an independent predictor for cardiovascular death (HR 2.75, 95% CI 1.10–6.83), although RV-RA pressure gradient trended towards significance (HR 1.01 per-1mmHg increase, 95% CI 1.00–1.03).

## Discussion

This retrospective observational study delineates the “real-world” inpatient use of TTE following admission for confirmed acute PE over a 12-year period. The key findings are: 1) low prevalent use of TTE during admission for acute PE, with the majority performed after day-1; 2) multivariable analysis suggested inpatient TTE use is site-dependent and is more likely performed in patients with a known history of cardiovascular disease and less likely in those with a history of malignancy; 3) though the use of inpatient TTE was not an independent predictor of mortality in this study, it is likely confounded by the fact that sicker patients were more likely to get the TTE. This is suggested by the observation that patients who had an inpatient TTE had longer hospital admission and significantly shorter survival time, consistent with the observation of higher sPESI scores indicative of higher risk patients. Additionally, 4) in the patients who had an inpatient TTE following acute PE, RV-RA pressure gradient was an independent predictor of prognosis.

TTE cannot be relied upon to diagnose PE, with reported sensitivity as low as 38% [6, 7]. Definitive TTE imaging demonstrating right heart or proximal pulmonary artery thrombus, reported in small case series [26], is uncommon and most often accompanies massive PE with high mortality. TTE is of most use in risk stratification; the prognostic value of RV dysfunction is recognized [9, 11], even in the absence of hemodynamic compromise [8]. TTE is also useful for excluding other critical differential diagnoses such as cardiac tamponade; PE as a cause of shock can be reliably excluded in the presence of a normal RV and should prompt investigation for another cause [13].

Our study demonstrates that clinical utilization of TTE is certainly not routine following acute PE, varying significantly between the two centers studied despite its recommendation in PE management guidelines. The main clinical drivers for its use were a prior history of cardiovascular disease, while patients with known malignancy were less likely to undergo inpatient TTE, the latter possibly reflecting a global view of the patient’s overall prognosis. We also observed that in the inpatient TTE group, the length of stay at both sites was significantly longer than those patients who did not received an inpatient TTE. Furthermore, patients at LH who underwent inpatient TTE had a significantly higher mortality than those who did not. This suggests selective clinical utilization of TTE for ‘unwell’ patients, particularly at LH. CRGH was an independent predictor of reduced all-cause and cardiovascular mortality, despite a more comorbid population. Taken together, this suggests that LH treated a more acutely unstable population. Indeed, the unadjusted in-hospital mortality at LH was two-fold that of CRGH. Although clinical markers of hemodynamic compromise were not available for the LH cohort, the greater prevalence of RV dilatation and higher mean RV-RA pressure gradient at LH supports this conclusion. Likewise, a sPESI of 0, indicating a low 30-day mortality approximating 1%, was identified in 41.2% of the CRGH cohort, suggesting an overall lower thrombotic burden.

Under these conditions of low and selective TTE use, neither the use nor timing of inpatient TTE were independent predictors of mortality. This may be explained by a number of factors. It can be presumed that for most patients, treatment, and therefore outcome, would not be altered based on TTE findings alone, other than in very unwell patients in shock or requiring intensive care. There were few of these critically unwell patients in the larger CRGH cohort, as reflected by the overall low sPESI. Only 11 patients at CRGH had a systolic blood pressure <90mmHg on presentation. This may have confounded any effects seen in the sicker, smaller LH cohort. Furthermore, as TTE was performed predominantly after the first 24 hours of admission, transient RV dysfunction—of known prognostic importance in acute PE—could be missed, negating the opportunity to institute earlier or more aggressive therapy and therefore diluting the impact of TTE timing. Additionally, patients with malignancy, known to have a higher mortality in acute PE [27], were under-represented in the TTE cohort. Overall, it is therefore difficult to draw conclusions regarding the impact of TTE on outcome.

The main limitations of our study are its retrospective design; unmeasured confounders may have influenced the analysis. Although all efforts were made to gather complete documentation for each patient, this was not possible for all patients, while additional hemodynamic and blood parameters were not available at LH, rendering more comprehensive assessment and comparison between the two cohorts difficult. In addition, as we only included patients with confirmed PE, the prevalence of TTE use in those who were suspected but did not have PE is not known. In the present study, about 70% of patients underwent V/Q scan to diagnose PE. Although the use of CTPA increased over the study 12-year period (data not shown), the more prevalent use of V/Q scan might have led to under-diagnosis, though in some instances, V/Q scan is more sensitive than CTPA especially for chronic thromboembolic disease. The main advantages of CTPA are its speed and easier interpretability by the majority of radiologists. The echocardiographic parameters were retrospectively extracted from semi-qualitative reports, and detailed echocardiographic parameters such as quantitative RV assessment and chronicity of abnormal findings were not available. These are important echocardiographic parameters to include in future study as the most common cause of early death in these patients is right ventricular failure. With overall small numbers in the LH-site TTE subgroup, relevant subgroup analyses must be interpreted with caution. Finally, we could not adjust for the impact of type, duration or time to institution of therapy, and any requirement for adjunctive hemodynamic support, and were not able to determine the rates of recurrent non-fatal PE.

### Conclusion

In this real-world, multicenter observational study of 2306 patients admitted with acute PE, the overall prevalence of inpatient TTE use was low. TTE is a valuable clinical tool and in real-world setting, risk stratification with TTE tends to be in higher risk PE patients. Although a class IIa recommendation, not every patient with a PE needs to have a TTE, especially if the thrombus burden is low and there is no evidence of RV dysfunction on CT scan (although the majority of patients in this study had V/Q). Future studies should identify patients that will benefit the most to have a TTE assessment following an acute PE episode, and explore strategies to reduce the barriers in accessing TTE in clinical practice.

## Supporting Information

S1 Dataset (XLSX)

S1 Fig Unadjusted Kaplan-Maier survival curves between patients who received an inpatient TTE versus no TTE during index PE admission (LH cohort). The unbroken line represents patients who did not received an inpatient transthoracic echocardiogram (TTE), whilst the broken line represents patients who had an inpatient TTE during the index admission for acute pulmonary embolism (PE). The curves differed significantly for the study period (*P* = 0.009). LH, Liverpool Hospital.
(TIF)

S2 Fig Unadjusted Kaplan-Maier survival curves between patients who received an inpatient TTE versus no TTE during index PE admission (CRGH cohort). The unbroken line represents patients who did not received an inpatient transthoracic echocardiogram (TTE), whilst the broken line represents patients who had an inpatient TTE during the index admission for acute pulmonary embolism (PE). The curves did not differ significantly for the study period (*P* = 0.31). CRGH, Concord Repatriation General Hospital.
(TIFF)

S1 Table Patient characteristics during index PE admission. (DOCX)

S2 Table Additional patient characteristics for CRGH. (DOCX)

S3 Table Univariable predictors for having an inpatient TTE. (DOCX)

S4 Table Multivariable predictors for having an inpatient TTE. (DOCX)

S5 Table All-cause and cardiovascular mortality after acute PE. (DOCX)

S6 Table Univariable predictors of all-cause mortality: combined cohort. (DOCX)

S7 Table Multivariable independent predictors of mortality (Model 1). (DOCX)

S8 Table Multivariable independent predictors of mortality: inpatient TTE subgroup (Model 2). (DOCX)

S9 Table Multivariable independent predictors of mortality: CRGH cohort only. (DOCX)

S10 Table Univariable echocardiographic predictors of mortality for inpatient TTE subgroup of combined cohort. (DOCX)
