# Supplementary figures and images for: Prevalence of Echocardiography Use in Patients Hospitalized with Confirmed Acute Pulmonary Embolism: A Real-World Observational Multicenter Study
Source: PLoS One. 2016 Dec 15;11(12):e0168554. doi: 10.1371/journal.pone.0168554 (PMC5158194; doi:10.1371/journal.pone.0168554)

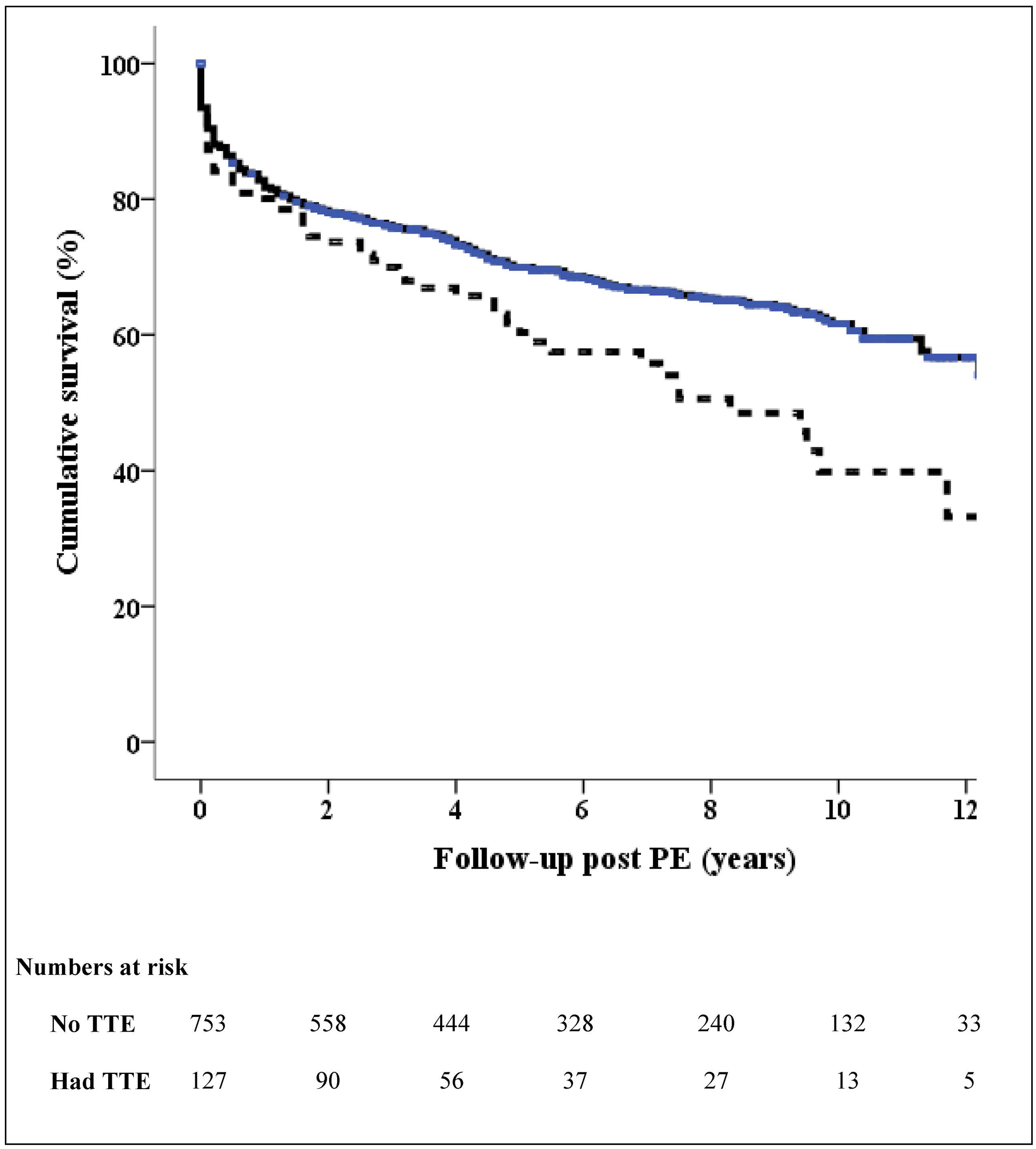

Supplement: S1 Fig — The unbroken line represents patients who did not received an inpatient transthoracic echocardiogram (TTE), whilst the broken line represents patients who had an inpatient TTE during the index admission for acute pulmonary embolism (PE). The curves differed significantly for the study period (P = 0.009). LH, Liverpool Hospital. (TIF) [file pone.0168554.s002.tif]

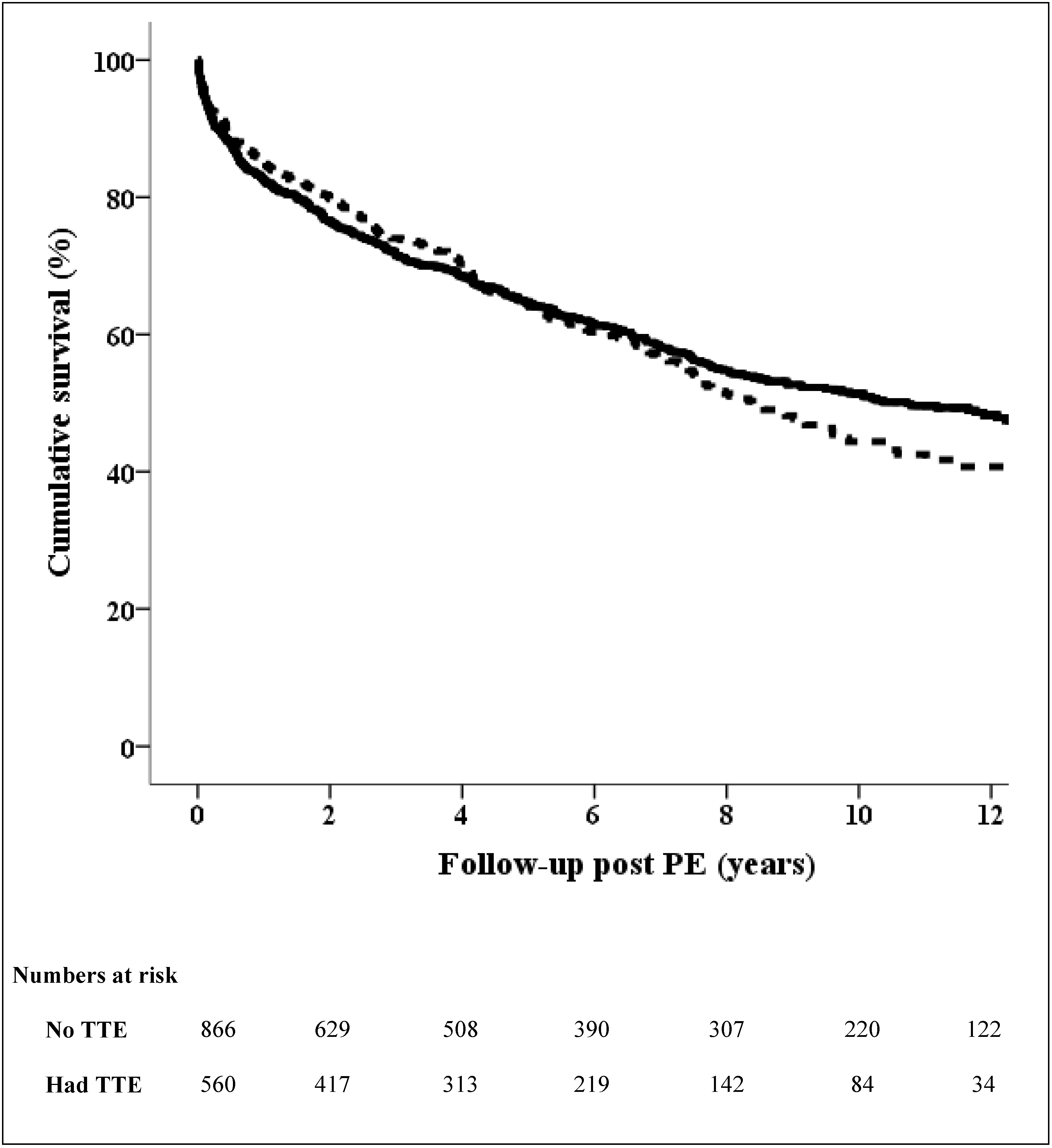

Supplement: S2 Fig — The unbroken line represents patients who did not received an inpatient transthoracic echocardiogram (TTE), whilst the broken line represents patients who had an inpatient TTE during the index admission for acute pulmonary embolism (PE). The curves did not differ significantly for the study period (P = 0.31). CRGH, Concord Repatriation General Hospital. (TIFF) [file pone.0168554.s003.tiff]
