# Supplementary material for: Prevalence of Echocardiography Use in Patients Hospitalized with Confirmed Acute Pulmonary Embolism: A Real-World Observational Multicenter Study
Source: PLoS One. 2016 Dec 15;11(12):e0168554. doi: 10.1371/journal.pone.0168554 (PMC5158194; doi:10.1371/journal.pone.0168554)
Supplement: S1 Table — (DOCX) [file pone.0168554.s004.docx]

**S1 Table. Patient characteristics during index PE admission.**

|  | **CRGH** | | | **LH** | | |
| --- | --- | --- | --- | --- | --- | --- |
|  | **+TTE** | **-TTE** | **Total** | **+TTE** | **-TTE** | **Total** |
| **Admission parameters** | **n=560** | **n=866** | **n=1426** | **n=127** | **n=753** | **n=880** |
| Age – years | 69.6±15.2* | 66.2±16.9 | 67.5±16.4 | 62.3±17.3^†^ | 59.5±17.4 | 59.9±17.4 |
| Males – no. (%) | 248 (44.3) | 381 (44.0) | 629 (44.1) | 58 (45.7) | 332 (44.1) | 390 (44.3) |
| Admission length – days | 9.7±7.3* | 6.8±5.9 | 7.9±6.6 | 11.8±8.3^†^ | 7.5±7.0 | 8.1±7.4 |
| Days to inpatient TTE | 3.0±6.7 | - | - | 4.8±4.9 | - | - |
| Early TTE – no. (%) | 208 (37.1) | - | - | 34 (27) | - | - |
| **Imaging** **– no. (%)** |  |  |  |  |  |  |
| V/Q scintigraphy | 416 (74.3) | 659 (76.1) | 1075 (75.4) | 55 (43.3) | 308 (40.9) | 363 (41.3) |
| CTPA | 207 (37.0) | 287 (33.1) | 494 (34.6) | 63 (49.6) | 341 (45.3) | 404 (45.9) |
| **Comorbidities – no. (%)** |  |  |  |  |  |  |
| Cardiovascular disease | 279 (49.8)* | 267 (30.8) | 546 (38.3) | 49 (38.6)^†^ | 153 (20.3) | 202 (23.0) |
| IHD | 118 (21.1)* | 118 (13.6) | 236 (16.5) | 20 (15.7) | 79 (10.5) | 99 (11.3) |
| CCF | 100 (17.9)* | 59 (6.8) | 159 (11.2) | 10 (7.9) | 46 (6.1) | 56 (6.4) |
| Atrial fibrillation/flutter | 109 (19.5)* | 88 (10.2) | 197 (13.8) | 12 (9.4) | 49 (6.5) | 61 (6.9) |
| Valvular heart disease | 16 (2.9) | 18 (2.1) | 34 (2.4) | 4 (3.1) | 17 (2.3) | 21 (2.4) |
| Stroke | 15 (2.7) | 25 (2.9) | 40 (2.8) | 2 (1.6) | 10 (1.3) | 12 (1.4) |
| PVD | 66 (11.8) | 77 (8.9) | 143 (10.0) | 6 (4.7) | 30 (4.0) | 36 (4.1) |
| Cardiac risk factors | 308 (55)* | 410 (47.3) | 718 (50.4) | 89 (70.1) | 460 (61.1) | 549 (62.4) |
| Hypertension | 166 (29.6)* | 188 (21.7) | 354 (24.8) | 36 (28.3) | 199 (26.4) | 235 (26.7) |
| Dyslipidemia | 71 (12.7)* | 81 (9.4) | 152 (10.7) | 20 (15.7) | 87 (11.6) | 107 (12.2) |
| Diabetes | 91 (16.3)* | 100 (11.5) | 191 (13.4) | 32 (25.2)^†^ | 99 (13.1) | 131 (14.9) |
| Current smoker | 40 (7.1) | 77 (8.9) | 117 (8.2) | 24 (18.9) | 155 (20.6) | 179 (20.3) |
| Ex-smoker | 104 (18.6) | 142 (16.4) | 246 (17.3) | 16 (12.6) | 131 (17.4) | 147 (16.7) |
| Chronic pulmonary disease | 63 (11.3) | 111 (12.8) | 174 (12.2) | 13 (10.2) | 70 (9.3) | 83 (9.4) |
| Chronic kidney disease | 42 (7.5)* | 37 (4.3) | 79 (5.5) | 14 (11.0) | 56 (7.4) | 70 (8.0) |
| Malignancy | 87 (15.5)* | 214 (24.7) | 301 (21.1) | 18 (14.2) | 139 (18.5) | 157 (17.8) |
| CCI |  |  |  |  |  |  |
| Mean score | 1.56±1.86 | 1.65±2.14 | 1.62±2.03 | 1.24±1.43 | 1.03±1.22 | 1.06±1.25 |
| ≥ 3 | 128 (22.9) | 206 (23.8) | 334 (23.4) | 23 (18.1) | 94 (12.5) | 117 (13.3) |
| 1-2 | 226 (40.4)* | 284 (32.8) | 510 (35.8) | 52 (40.9) | 308 (40.9) | 360 (40.9) |
| 0 | 206 (36.8) | 376 (43.4) | 582 (40.8) | 52 (40.9) | 351 (46.6) | 403 (45.8) |

- *P*<0.05 between inpatient and no inpatient TTE patients at CRGH.
- *P*<0.05 between inpatient and no inpatient TTE patients at LH.

Plus-minus values represent mean ± standard deviation (all others represent numbers of patients with values in brackets representing percentages).

The abbreviations +TTE and –TTE denote patients who did and did not undergo inpatient TTE respectively; CRGH, Concord Repatriation General Hospital; LH, Liverpool Hospital; IHD, ischemic heart disease; CCF, congestive cardiac failure; PVD, peripheral vascular disease; CCI, Charlson Comorbidity Index; V/Q, ventilation/perfusion; CTPA, computed tomography pulmonary angiography; TTE, transthoracic echocardiogram.
