# Supplementary material for: Prevalence of Echocardiography Use in Patients Hospitalized with Confirmed Acute Pulmonary Embolism: A Real-World Observational Multicenter Study
Source: PLoS One. 2016 Dec 15;11(12):e0168554. doi: 10.1371/journal.pone.0168554 (PMC5158194; doi:10.1371/journal.pone.0168554)
Supplement: S2 Table — (DOCX) [file pone.0168554.s005.docx]

**S2 Table. Additional patient characteristics for CRGH.**

| **Parameters** | **Inpatient TTE**  **n=560** | **No inpatient TTE n=866** |
| --- | --- | --- |
| Symptoms at presentation – no. (%) |  |  |
| Syncope | 48 (8.6)* | 34 (3.9) |
| Chest pain | 310 (55.4) | 461 (53.2) |
| Dyspnea | 397 (70.9)* | 542 (62.6) |
| Hemodynamics at presentation |  |  |
| Systolic blood pressure – mmHg | 142.0±26.7* | 138.9±22.9 |
| Systolic blood pressure <100mmHg – no. (%) | 20 (3.6) | 24 (2.8) |
| Heart rate – bpm | 90.6±22.0* | 86.8±20.4 |
| Heart rate >110 bpm – no. (%) | 81 (14.5)* | 91 (10.5) |
| Shock Index >0.7 – no. (%) | 180 (32.1) | 241 (27.8The ) |
| sPESI | 0.96±0.97* | 0.83±0.86 |
| sPESI >0 – no. (%) | 339 (60.5) | 499 (57.6) |
| Troponin-T performed – no. (%) | 382 (68.2)* | 397 (45.8) |
| >0.01mcg/L | 169 (30.2)* | 123 (14.2) |

- *P*<0.05 between inpatient and no inpatient TTE patients

Plus-minus values represent mean ± standard deviation (all others represent numbers of patients with values in brackets representing percentages).

Shock index is derived from heart rate divided by systolic blood pressure, with a score >0.7 signifying hemodynamic compromise; sPESI, simplified Pulmonary Embolism Severity Index; INR, international normalized ratio; TTE, transthoracic echocardiogram.
