# Supplementary material for: Prevalence of Echocardiography Use in Patients Hospitalized with Confirmed Acute Pulmonary Embolism: A Real-World Observational Multicenter Study
Source: PLoS One. 2016 Dec 15;11(12):e0168554. doi: 10.1371/journal.pone.0168554 (PMC5158194; doi:10.1371/journal.pone.0168554)
Supplement: S3 Table — (DOCX) [file pone.0168554.s006.docx]

**S3 Table. Univariable predictors for having an inpatient TTE.**

| **Admission parameters** | **Odds ratio (95% confidence interval)** | ***P* value** |
| --- | --- | --- |
| Site (CRGH) | 3.83 (3.10 – 4.76) | <0.001 |
| Age – per-1-year increase | 1.02 (1.01 – 1.02) | <0.001 |
| Male | 1.02 (0.85 – 1.22) | 0.82 |
| CTPA | 0.92 (0.77 – 1.11) | 0.39 |
| **Comorbidities** |  |  |
| Ischaemic heart disease | 1.81 (1.43 – 2.30) | <0.001 |
| Congestive cardiac failure | 2.75 (2.07 – 3.65) | <0.001 |
| Atrial fibrillation/flutter | 2.31 (1.78 – 3.01) | <0.001 |
| Valvular heart disease | 1.36 (0.78 – 2.37) | 0.28 |
| Stroke | 1.15 (0.64 – 2.06) | 0.64 |
| Peripheral vascular disease | 1.65 (1.21 – 2.26) | 0.002 |
| Hypertension | 1.32 (1.09 – 1.62) | 0.01 |
| Dyslipidemia | 1.32 (1.00 – 1.73) | 0.046 |
| Diabetes | 1.56 (1.22 – 1.99) | <0.001 |
| Current smoker | 0.61 (0.46 – 0.82) | 0.001 |
| Ex-smoker | 1.04 (0.82 – 1.32) | 0.72 |
| Chronic pulmonary disease | 0.99 (0.74 – 1.31) | 0.94 |
| Chronic kidney disease | 1.46 (1.03 – 2.06) | 0.03 |
| Malignancy | 0.65 (0.51 – 0.82) | <0.001 |
| CCI – per-1-score increase | 1.04 (0.99 – 1.09) | 0.10 |

CRGH, Concord Repatriation General Hospital; CCI, Charlson Comorbidity Index; CTPA, computed tomography pulmonary angiography
