# Supplementary material for: Prevalence of Echocardiography Use in Patients Hospitalized with Confirmed Acute Pulmonary Embolism: A Real-World Observational Multicenter Study
Source: PLoS One. 2016 Dec 15;11(12):e0168554. doi: 10.1371/journal.pone.0168554 (PMC5158194; doi:10.1371/journal.pone.0168554)
Supplement: S4 Table — (DOCX) [file pone.0168554.s007.docx]

**S4 Table. Multivariable predictors for having an inpatient TTE.**

| **Multivariable analysis *** | **Odds ratio (95% CI)** | ***P* value** |
| --- | --- | --- |
| Site (CRGH vs LH) | 3.60 (2.87 – 4.52) | <0.001 |
| Age – per-1-year increase | 1.01 (1.00 – 1.01) | 0.04 |
| Congestive cardiac failure | 1.86 (1.35 – 2.55) | <0.001 |
| Atrial fibrillation/flutter | 1.58 (1.18 – 2.11) | 0.002 |
| Diabetes | 1.44 (1.09 – 1.90) | 0.01 |
| Malignancy | 0.58 (0.45 – 0.75) | <0.001 |

- The univariables including sex, ischemic heart disease, peripheral vascular disease, hypertension, dyslipidaemia, current smoker and chronic kidney disease were not significant in the multivariable analysis.

CRGH, Concord Repatriation General Hospital; LH, Liverpool Hospital; TTE, transthoracic echocardiogram; CI, confidence interval.
