# Supplementary material for: Prevalence of Echocardiography Use in Patients Hospitalized with Confirmed Acute Pulmonary Embolism: A Real-World Observational Multicenter Study
Source: PLoS One. 2016 Dec 15;11(12):e0168554. doi: 10.1371/journal.pone.0168554 (PMC5158194; doi:10.1371/journal.pone.0168554)
Supplement: S5 Table — (DOCX) [file pone.0168554.s008.docx]

**S5 Table. All-cause and cardiovascular mortality after acute PE.**

|  | | | | | | |
| --- | --- | --- | --- | --- | --- | --- |
|  | **Combined cohort** | | **CRGH** | | **LH** | |
|  | **Inpatient +TTE** | **No inpatient - TTE** | **+TTE** | **- TTE** | **+TTE** | **- TTE** |
|  | **N=687** | **N=1619** | **n=560** | **n=866** | **n=127** | **n=753** |
| **All-cause mortality** | |  |  |  |  |  |
| In-hospital | 27 (3.9) | 74 (4.6) | 17 (3.0) | 28 (3.2) | 10 (7.9) | 46 (6.1) |
| 1-month | 35 (5.1) | 97 (6.0) | 23 (4.1) | 42 (4.8) | 12 (9.4) | 55 (7.3) |
| 3-month | 62 (9.0) | 173 (10.7) | 42 (7.5) | 81 (9.4) | 20 (15.7) | 92 (12.2) |
| 6-month | 86 (12.5) | 220 (13.6) | 63 (11.3) | 106 (12.2) | 23 (18.1) | 114 (15.1) |
|  |  |  |  |  |  |  |
| **Total mortality** | 308 (44.8) | 645 (39.8) | 251 (44.8) | 386 (44.6) | 57 (44.9) ^‡^ | 259 (34.4) |
| **CV mortality** | 125 (18.2) ^†^ | 232 (14.3) | 102 (18.2) | 133 (15.4) | 23 (18.1) | 99 (13.1) |
|  |  |  |  |  |  |  |
| Follow-up – months | 60.7±45.6 ^*^ | 68.9±50.8 | 62.3±45.8 ^*^ | 70.9±53.5 | 53.5±44.4 ^*^ | 66.5±47.5 |
|  |  |  |  |  |  |  |

- *P*<0.05 between inpatient and no inpatient TTE in each cohort.
- *P*<0.05 between inpatient and no inpatient TTE in combined cohort.
- *P*<0.05 between inpatient and no inpatient TTE in LH cohort.

Plus-minus values represent mean ± standard deviation (all others represent numbers of patients with values in brackets representing percentages).

CRGH, Concord Repatriation General Hospital; LH, Liverpool Hospital; TTE, transthoracic echocardiogram; CV, cardiovascular.
