# Supplementary material for: Prevalence of Echocardiography Use in Patients Hospitalized with Confirmed Acute Pulmonary Embolism: A Real-World Observational Multicenter Study
Source: PLoS One. 2016 Dec 15;11(12):e0168554. doi: 10.1371/journal.pone.0168554 (PMC5158194; doi:10.1371/journal.pone.0168554)
Supplement: S6 Table — (DOCX) [file pone.0168554.s009.docx]

**S6 Table. Univariable predictors of all-cause mortality: combined cohort.**

| **Admission parameters** | **Hazard ratio (95% confidence interval)** | ***P* value** |
| --- | --- | --- |
| Site (CRGH) | 1.18 (1.03 – 1.35) | 0.02 |
| Age – per-1-year increase | 1.04 (1.05 – 1.06) | <0.001 |
| Male | 1.28 (1.13 – 1.45) | <0.001 |
| Inpatient TTE | 1.22 (1.06 – 1.39) | 0.01 |
| **Comorbidities** |  |  |
| Ischaemic heart disease | 1.70 (1.45 – 1.99) | <0.001 |
| Congestive cardiac failure | 1.89 (1.57 – 2.27) | <0.001 |
| Atrial fibrillation/flutter | 1.75 (1.47 – 2.09) | <0.001 |
| Valvular heart disease | 1.14 (0.75 – 1.74) | 0.55 |
| Stroke | 1.58 (1.09 – 2.29) | 0.02 |
| Peripheral vascular disease | 1.32 (1.07 – 1.64) | 0.01 |
| Hypertension | 1.13 (0.98 – 1.30) | 0.10 |
| Dyslipidemia | 0.90 (0.74 – 1.10) | 0.30 |
| Diabetes | 1.34 (1.14 – 1.59) | 0.001 |
| Current smoker | 0.57 (0.45 – 0.71) | <0.001 |
| Ex-smoker | 1.12 (0.95 – 1.32) | 0.19 |
| Chronic pulmonary disease | 1.54 (1.29 – 1.85) | <0.001 |
| Chronic kidney disease | 2.38 (1.93 – 2.92) | <0.001 |
| Malignancy | 4.00 (3.50 – 4.57) | <0.001 |
| CCI – per-1-score increase | 1.39 (1.35 – 1.43) | <0.001 |

CRGH, Concord Repatriation General Hospital; CCI, Charlson Comorbidity Index.
