# Supplementary material for: Prevalence of Echocardiography Use in Patients Hospitalized with Confirmed Acute Pulmonary Embolism: A Real-World Observational Multicenter Study
Source: PLoS One. 2016 Dec 15;11(12):e0168554. doi: 10.1371/journal.pone.0168554 (PMC5158194; doi:10.1371/journal.pone.0168554)
Supplement: S7 Table — (DOCX) [file pone.0168554.s010.docx]

**S7 Table. Multivariable independent predictors of mortality (Model 1).**

|  | **All-cause mortality** | | **Cardiovascular mortality** | |
| --- | --- | --- | --- | --- |
| **Variables** | **HR (95% CI)** | ***P* value** | **HR (95% CI)** | ***P* value** |
| Inpatient TTE | 1.00 (0.87 – 1.16) | 0.98 | 0.99 (0.78 – 1.25) | 0.92 |
| Site (CRGH) | 0.73 (0.63 – 0.84) | <0.001 | 0.70 (0.55 – 0.89) | 0.004 |
| Age – per-1year increase | 1.05 (1.05 – 1.06) | <0.001 | 1.07 (1.06 – 1.08) | <0.001 |
| Male | 1.45 (1.28 – 1.65) | <0.001 | 1.57 (126 – 1.94) | <0.001 |
| Ischaemic heart disease | 1.14 (0.96 – 1.34) | 0.13 | 1.30 (1.01 – 1.69) | 0.045 |
| Congestive cardiac failure | 1.29 (1.06 – 1.57) | 0.01 | 1.60 (1.20 – 2.13) | 0.002 |
| Atrial fibrillation/flutter | 1.09 (0.91 – 1.31) | 0.36 | 1.27 (0.97 – 1.66) | 0.08 |
| Stroke | 1.26 (0.87 – 1.83) | 0.23 | 1.36 (0.79 – 2.33) | 0.27 |
| Peripheral vascular disease | 1.09 (0.88 – 1.36) | 0.44 | 1.30 (0.94 – 1.80) | 0.11 |
| Diabetes | 1.07 (0.90 – 1.26) | 0.46 | 1.23 (0.94 – 1.61) | 0.13 |
| Current smoker | 0.95 (0.75 – 1.20) | 0.64 | 1.11 (0.76 – 1.61) | 0.59 |
| Chronic pulmonary disease | 1.39 (1.16 – 1.68) | <0.001 | 1.24 (0.90 – 1.70) | 0.19 |
| Chronic kidney disease | 1.46 (1.18 – 1.81) | <0.001 | 2.13 (1.55 – 2.93) | <0.001 |
| Malignancy | 3.49 (3.05 – 3.99) | <0.001 | 2.38 (1.84 – 3.07) | <0.001 |

CRGH, Concord Repatriation General Hospital; CI, confidence interval; HR, hazard ratio; TTE, transthoracic echocardiogram.
