# Supplementary material for: Prevalence of Echocardiography Use in Patients Hospitalized with Confirmed Acute Pulmonary Embolism: A Real-World Observational Multicenter Study
Source: PLoS One. 2016 Dec 15;11(12):e0168554. doi: 10.1371/journal.pone.0168554 (PMC5158194; doi:10.1371/journal.pone.0168554)
Supplement: S8 Table — (DOCX) [file pone.0168554.s011.docx]

**S8 Table. Multivariable independent predictors of mortality: inpatient TTE subgroup (Model 2).**

|  | **All-cause mortality** | | **Cardiovascular mortality** | |
| --- | --- | --- | --- | --- |
| **Variables** | **HR (95% CI)** | ***P* value** | **HR (95% CI)** | ***P* value** |
| Early (day-0 or 1) inpatient TTE | 1.07 (0.83 – 1.37) | 0.61 | 1.15 (0.79 – 1.67) | 0.47 |
| Site (CRGH) | 0.57 (0.43 – 0.77) | <0.001 | 0.60 (0.37 – 0.95) | 0.03 |
| Age – per-1year increase | 1.04 (1.03 – 1.05) | <0.001 | 1.05 (1.04 – 1.07) | <0.001 |
| Male | 1.28 (1.02 – 1.61) | 0.04 | 1.38 (0.96 – 2.00) | 0.09 |
| CCI – per-1-score increase | 1.30 (1.23 – 1.37) | <0.001 | 1.38 (1.27 – 1.51) | <0.001 |

CRGH, Concord Repatriation General Hospital; CCI, Charlson Comorbidity Index; CI, confidence interval; HR, hazard ratio; TTE, transthoracic echocardiogram.
