# Supplementary material for: Prevalence of Echocardiography Use in Patients Hospitalized with Confirmed Acute Pulmonary Embolism: A Real-World Observational Multicenter Study
Source: PLoS One. 2016 Dec 15;11(12):e0168554. doi: 10.1371/journal.pone.0168554 (PMC5158194; doi:10.1371/journal.pone.0168554)
Supplement: S9 Table — (DOCX) [file pone.0168554.s012.docx]

**S9 Table. Multivariable independent predictors of mortality: CRGH cohort only.**

|  | **All-cause mortality** | | **Cardiovascular mortality** | |
| --- | --- | --- | --- | --- |
| **Variables** | **HR (95% CI)** | ***P* value** | **HR (95% CI)** | ***P* value** |
| Inpatient TTE | 0.84 (0.71 – 0.99) | 0.04 | 0.87 (0.66 – 1.14) | 0.31 |
| V/Q scintigraphy | 0.44 (0.34 – 0.59) | <0.001 | 0.69 (0.50 – 0.94) | 0.02 |
| CTPA* | 0.81 (0.62 – 1.06) | 0.12 | - | - |
| Male | 1.28 (1.09 – 1.50) | 0.002 | 1.19 (0.91 – 1.55) | 0.20 |
| Ischaemic heart disease | 1.46 (1.22 – 1.76) | <0.001 | 2.25 (1.69 – 3.00) | <0.001 |
| Atrial fibrillation/flutter | 1.10 (0.90 – 1.35) | 0.36 | 1.47 (1.08 – 2.02) | 0.02 |
| Stroke | 1.24 (0.84 – 1.83) | 0.29 | 1.55 (0.89 – 2.69) | 0.13 |
| Peripheral vascular disease | 1.25 (0.99 – 1.57) | 0.07 | 1.49 (1.05 – 2.11) | 0.03 |
| Diabetes | 1.02 (0.82 – 1.23) | 0.83 | 1.14 (0.79 – 1.64) | 0.48 |
| Current smoker | 0.67 (0.48 – 0.94) | 0.02 | 0.71 (0.42 – 1.19) | 0.19 |
| Chronic kidney disease | 1.69 (1.27 – 2.24) | <0.001 | 2.45 (1.57 – 3.84) | <0.001 |
| sPESI – per-1-score increase | 1.93 (1.79 – 2.10) | <0.001 | 2.04 (1.78 – 2.33) | <0.001 |
| Troponin-T >0.01mcg/L | 1.20 (0.99 – 1.45) | 0.07 | 1.48 (1.09 – 2.02) | 0.01 |

- CTPA was not a univariable predictor for cardiovascular mortality.

CRGH, Concord Repatriation General Hospital; CCI, Charlson comorbidity index; CI, confidence interval; CTPA, computed tomography pulmonary angiography; V/Q, ventilation/perfusion; HR, hazard ratio; TTE, transthoracic echocardiogram; sPESI, simplified Pulmonary Embolism Severity Index.
