# Supplementary material for: Prevalence of Echocardiography Use in Patients Hospitalized with Confirmed Acute Pulmonary Embolism: A Real-World Observational Multicenter Study
Source: PLoS One. 2016 Dec 15;11(12):e0168554. doi: 10.1371/journal.pone.0168554 (PMC5158194; doi:10.1371/journal.pone.0168554)
Supplement: S10 Table — (DOCX) [file pone.0168554.s013.docx]

**S10 Table. Univariable echocardiographic predictors of mortality for inpatient TTE subgroup of combined cohort.**

|  | **All-cause mortality** | | **Cardiovascular mortality** | |
| --- | --- | --- | --- | --- |
| **Variables** | **HR (95% CI)** | ***P* value** | **HR (95% CI)** | ***P* value** |
| LV ejection fraction ≤50% | 1.53 (1.21 – 1.92) | <0.001 | 2.38 (1.68 – 3.39) | <0.001 |
| RV dilatation | 1.27 (0.98 – 1.66) | 0.07 | 1.27 (0.89 – 1.83) | 0.19 |
| Impaired RV contractility | 1.61 (1.28 – 2.03) | <0.001 | 1.86 (1.31 – 2.66) | 0.001 |
| RV-RA pressure gradient – 1mmHg increase | 1.02 (1.02 – 1.03) | <0.001 | 1.03 (1.02 – 1.04) | <0.001 |
| LA dilatation | 1.57 (1.24 – 1.98) | <0.001 | 2.09 (1.45 – 3.01) | <0.001 |
| RA dilatation | 1.48 (1.18 – 1.88) | 0.001 | 1.68 (1.18 – 2.41) | 0.004 |
| Valvular lesions (moderate/severe) |  |  |  |  |
| Aortic stenosis | 3.75 (2.10 – 6.69) | <0.001 | 5.15 (2.40 – 11.1) | <0.001 |
| Aortic regurgitation | 1.71 (0.85 – 3.46) | 0.13 | 2.23 (0.71 – 7.02) | 0.17 |
| Mitral stenosis | 2.08 (0.67 – 6.48) | 0.21 | 1.94 (0.27 – 13.9) | 0.51 |
| Mitral regurgitation | 1.68 (1.17 – 2.37) | 0.004 | 2.71 (1.70 – 4.34) | <0.001 |
| Tricuspid regurgitation | 1.90 (1.44 – 2.51) | <0.001 | 2.56 (1.70 – 3.84) | <0.001 |

HR, hazard ratio; CI, confidence interval; LV, left ventricle; RV, right ventricle; LA, left atrium; RA, right atrium; EF, ejection fraction; TTE, transthoracic echocardiogram.
